# Comparison of Coated and Immobilized Chiral Stationary Phases Based on Amylose tris-[(*S*)-α-Methylbenzylcarbamate] for the HPLC Enantiomer Separation of α-Lipoic Acid and Its Reduced Form

**DOI:** 10.3390/molecules26061747

**Published:** 2021-03-20

**Authors:** Alessia Rosetti, Claudio Villani, Marco Pierini, Roberto Cirilli

**Affiliations:** 1 Centre for the Control and Evaluation of Medicines, Chemical Medicines Unit, Istituto Superiore di Sanità, Viale Regina Elena 299, 00161 Rome, Italy; alessia.rosetti@iss.it; 2 Department of Chemistry and Technology of Drugs, Sapienza University of Rome, 00185 Rome, Italy; claudio.villani@uniroma1.it (C.V.); marco.pierini@uniroma1.it (M.P.)

**Keywords:** amylose-based CSPs, chiralpal AS-H, chiralpak IH-3, enantioselective HPLC, α-lipoic acid, α-dihydrolipoic acid, absolute configuration

## Abstract

The couple of chiral sulfur compounds α-lipoic acid (ALA)/α-dihydrolipoic acid (DHALA) has attracted considerable attention in recent years owing to its remarkable anti-inflammatory and antioxidant properties. It is well known that the chirality of the C6 plays a key role in determining the biological activity of ALA. The natural occurring (*R*)-ALA enantiomer is an essential cofactor for key oxidative metabolism enzyme complexes and, after oral administration of the racemic mixture, it shows higher plasma concentration than (*S*)-ALA. Differently, the in vivo enantioselective action difference between the enantiomers of DHALA has not yet been studied. This lacking is perhaps due to the unavailability of analytical methods capable of determining the enantiomeric composition of biological samples during pharmacokinetic and pharmacodynamic events. In the present work, the direct and baseline enantioresolution of both chiral acids by HPLC on two amylose-derived chiral stationary phases is presented. The proposed chiral enantioselective protocol, therefore, does not require pre- or on-column derivatization. The performance of the coated Chiralpak AS-H CSP and the new immobilized Chiralpak IH-3 CSP, which have the same chiral selector amylose tris-[(*S*)-α-methylbenzylcarbamate], were compared using conventional normal-phase mobile phases containing ethanol or 2-propanol as alcoholic solvents and a fixed percentage of trifluoroacetic acid. Nonconventional eluents containing dichloromethane, ethyl acetate, and 2-methyltetrahydrofuran as organic cosolvents were applied in the separation of the enantiomers of two carboxylic acids on the immobilized Chiralpak IH-3 CSP. The effect of the column temperature was carefully evaluated in order to improve enantioselectivity. Adequate amounts of enantiomers were isolated by an analytical-size Chiralpak IH-3 column and submitted to chiroptical measurements. The absolute configuration assignment of the isolated enantiomers was determined by a multidisciplinary procedure based on the comparison of the experimental and calculated chiroptical properties.

## 1. Introduction

**Figure 1:**
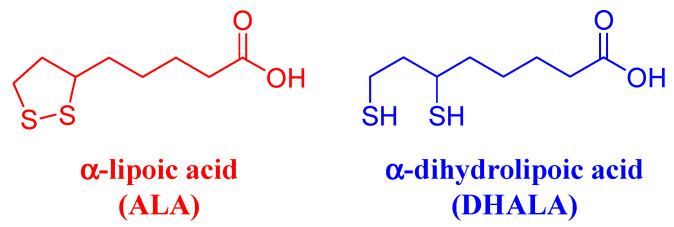
Chemical structures and abbreviations of α-lipoic acid and α-dihydrolipoic acid.

α-lipoic acid (Figure 1) (ALA), known also as 6,8-thioctic acid, is a chiral disulphide-containing compound synthesized enzymatically in human mitochondria from cysteine and octanoic acid. ALA contains a stereogenic C6 atom, and thus it exists as (*R*)- and (*S*)-enantiomers.

The (*R*)-enantiomer (R-ALA) is the endogenously synthesized form, which acts as cofactor for mitochondrial enzyme complexes involved in energy production such as those of pyruvate dehydrogenase and glycine decarboxylase [1].

Beyond its physiological functions, ALA is attracting growing interest in terms of research and pharmaceutical application as an antioxidant agent in preventive medicine, especially for age-related disorders such as cancer, neurological problems, and heart diseases. Exogenous ALA is partially reduced in vivo into α-dihydrolipoic acid (DHALA) (Figure 1). The DHALA/ALA has a very negative redox potential (i.e., −320 mV), which is responsible for its powerful reductive character [2]. ALA/DHALA antioxidant pair is capable of directly scavenging numerous toxic radical species, which can form in cellular metabolic processes for energy production (e.g., hydroxyl radical, peroxyl radical, oxygen singlet), protecting the cell from oxidative damage resulting from exposure to these substances [1]. DHALA is able to donate its two electrons to the oxidized and therefore inactive forms of glutathione (glutathione disulfide) and vitamin C (radical of vitamin C), regenerating them to glutathione and ascorbate. In turn, vitamin C in reduced form is able to reactivate the radical form of vitamin E reducing it to tocopherol (active vitamin E) [3].

Furthermore, clinical evidence suggests that ALA is also able: (i) to effectively chelate various heavy metals such as copper, manganese, zinc, calcium, mercury, and cadmium, limiting their toxicity [4] and (ii) to reduce blood glucose levels and to enhance insulin sensitivity in patients with diabetes [5].

In vivo studies indicate that R-ALA is more efficiently absorbed, and its bioavailability is significantly higher than S-ALA, independent from the type of pharmaceutical formulation used [6,7]. Thus, R-ALA would be preferred to the racemic form as a drug or dietary supplement. It is worth noting that, despite the achieved knowledge about pharmacokinetic and pharmacodynamics properties of the enantiomers of ALA, the role played by chirality in the biological events involving DHALA is obscure. This is perhaps due to the lack of studies on enantiomer separation of DHALA and/or the poor availability of commercially available enantiomeric forms.

Racemic ALA has been resolved by Fleischhauer et al. using an indirect reversed-phase liquid chromatography (RPLC) method involving a precolumn chemical derivatization with o-phthalaldehyde in the presence of d-phenylalanine [8].

The direct resolution of ALA was achieved by Saito et al. using the coated amylose-based Chiralpak AD-3R chiral stationary phase (CSP) with a mobile phases composed of acetonitrile-methanol-formic acid (10 mM) 25:25:50 (*v*/*v*/*v*) [9]. The Chiralpak AD-RH column, which contains the same amylose tris(3,5-dimethylphenyl-carbamate) selector, has demonstrated chiral resolving ability toward ALA, also employing a gradient elution with water/methanol added of formic acid [10].

Here, we report on the capability of two alternative commercially available amylose-based CSPs (Chiralpak AS-H and Chiralpak IH-3 CSPs) to discriminate the enantiomers of ALA and DHALA under normal phase conditions. The influence of mobile phase composition and column temperature on enantioselectivity and enantiomer elution order have been carefully evaluated.

## 2. Results and Discussion

### 2.1. Enantioseparation under Normal-Phase Conditions

Chiralpak AS-H and Chiralpak IH-3 are commercially available CSPs based on amylose tris-[(*S*)-α-methylbenzylcarbamate]. The polymeric selector is physically adsorbed onto 5 μm silica particles in the Chiralpak AS-H CSP, and it is chemically immobilized onto 3 μm silica particles in the Chiralpak IH-3 CSP.

The coated-type Chiralpak AS-H CSP can operate enantiorecogniton in normal-phase and polar organic conditions, but because of the modality of anchoring of polymer to silica it can be used only with limited types of solvents such as hydrocarbons (typically n-hexane) and alcohols (2-propanol, ethanol, methanol) [11,12,13]. Other organic solvents such as dichloromethane (DCM) and ethyl acetate (EA) can dissolve or swell the amylose derived and consequently, irreversibly degrade the chromatographic support. The immobilized version of the Chiralpak AS-H CSP, the Chiralpak IH-3 CSP, is a new chiral packing material, which has been designed to overcome these drawbacks and to work without any restriction in the choice of the solvents used for the preparation of the mobile phase [14].

The chromatographic enantioseparations of ALA and DHALA were initially performed using 25 cm length columns packed with the aforementioned CSPs in standard normal-phase mode. To explore these conditions, the mobile phases n-hexane/2-propanol (IPA)/trifluoroacetic acid (TFA) 80:20:0.1 (*v*/*v*/*v*) and n-hexane/ethanol (EtOH)/TFA 85:15:0.1 (*v*/*v*/*v*) were selected. The presence of 0.1% TFA in the mobile phases was helpful to improve peak shape and efficiency.

Comparing the retention and enantioseparation factor values summarized in Table 1 it can be highlighted that:

(i) at 25 °C only ALA was baseline resolved (Rs > 1.5) on both CSPs, whereas the discrimination of the enantiomers of DHALA was very poor (i.e., the maximum value of α was 1.06);
(ii) the chiral separation ability of the CSPs toward ALA decreased using ethanol as an alcoholic modifier (for example, α was 1.11 with ethanol and 1.29 with IPA on the Chirapak AS-H CSP).

The enantioseparation of ALA and the complete solvent compatibility of the Chiralpak IH-3 CSP encouraged us to set a second series of enantioselection experiments based on the use of alternative mobile phases containing different percentages of IPA and non-standard cosolvents such as DCM, EA, tetrahydrofuran (THF), and 2-methyltetrahydrofuran (MTHF). The best performances in terms of retention and enantioseparation were obtained by setting the IPA content at 5% and adding 15% of DCM or THF, or 5% of EA. Analyzing the chromatographic data summarized in Table 1 can be noted that, among the non-standard eluent systems used, the mixture hexane/IPA/DCM/TFA 80:5:15:0.1 (*v*/*v*/*v*/*v*) gave the best enantioselectivity for both chiral acids. In particular, the resolution values of DHALA on the Chiralpak IH-3 were unexpectedly greater than those observed for ALA (i.e., 2.88 vs. 1.89). The Chiralpak AS-H CSP remained superior to the immobilized counterpart in the resolution of ALA (i.e., Rs = 3.14 with n-hexane/IPA/TFA 80:20:0.1 (*v*/*v*/*v*)).

The effects produced by the solvent MTHF on enantioselectivity deserves special mention. With increasing environmental awareness, chemists are challenged in the search for cleaner, greener, and more sustainable solvents.

Along this line, we selected MTHF as an alternative solvent to THF and DCM in the preparation of non-standard normal-phase mobile phases. In fact, MTHF is a solvent produced from renewable raw materials, it is biodegradable, and it has a promising toxicological properties [15,16]. Despite these characteristics, HPLC enantioseparations carried out with mobile phases containing MTHF have not yet been described in the literature.

Looking at the data of enantioseparation reported in Table 1, it is interesting to note that switching solvents gave promising results. In particular, a substantial improvement in resolution with respect to THF, especially in the case of DHALA, was observed. At 25 °C and using the mobile phase hexane/IPA/MTHF/TFA 80:5:15:0.1 (*v*/*v*/*v*/*v*), the resolution of DHALA was complete (Rs = 1.89), whereas the enantiomers of ALA were slightly resolved (Rs = 1.12).

### 2.2. Temperature-Variable HPLC

It is widely recognized that the column temperature is a critical parameter in enantioselective HPLC [17,18]. Thus, to optimize the enantioseparation of ALA and DHALA on the Chiralpak AS-H and Chiralpak IG-3 CSPs, a temperature-dependent study was carried out. The retention and enantioselectivity factor values were recorded between 5 °C and 45 °C in 10 °C increments using as mobile phases the standard mixture n-hexane/IPA/TFA 80:20:0.1 (*v*/*v*/*v*) with Chiralpak AS-H and Chiralpak IH-3 CSPs and the non-standard mixtures n-hexane/IPA/EA/TFA 90:5:5:0.1 (*v*/*v*/*v*/*v*), n-hexane/IPA/DCM/TFA 80:5:15:0.1 (*v*/*v*/*v*/*v*), and n-hexane/IPA/MTHF/TFA 80:5:15:0.1 (*v*/*v*/*v*/*v*) with the immobilized Chiralpak IH-3 CSP.

The thermodynamic parameters governing the resolution process were calculated by correlating the enantioselectivity factors to temperature according to the following equation:

ln α = −ΔΔH°/RT + ΔΔS°/R(1)

where ΔΔH° and ΔΔS° are the differences in enthalpy and entropy of adsorption of the more and less retained enantiomers onto stationary phase, R is the gas constant, and T the absolute temperature.

As previously reported for enantioseparations dominated by a unique chiral recognition mechanism [19], the ln α vs. 1/T × 10^3^ van’t Hoff plots followed a linear pattern (*R*^2^ was always greater than 0.98).

In Table 2 are summarized the enantioseparation and resolution factors obtained at 5 °C and the thermodynamic parameters calculated by van’t Hoff analysis (slope = −ΔΔH°/R and intercept = ΔΔS°/R of the ln α vs. 1/T × 10^3^ plots).

Table 2 also provides the values of the temperature of isoenantioselectivity, T_ISO_ (i.e., temperature at which α = 1) [20,21,22,23], calculated according to Equation (2):

T_ISO_ = ΔΔH°/ΔΔS° (2)

**Figure 2:**
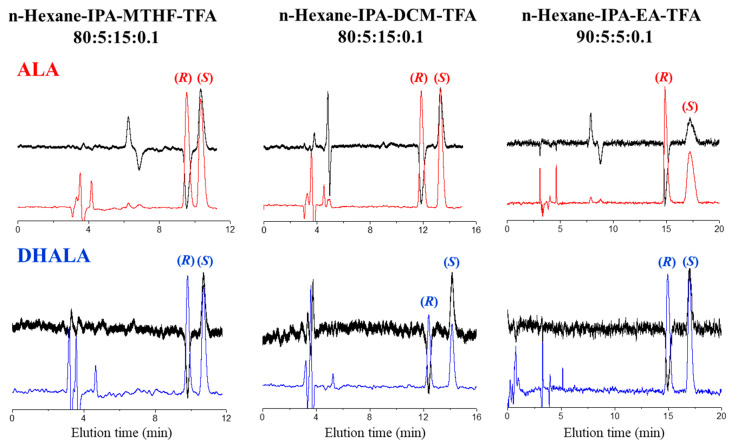
Chromatograms illustrating the impact of non-standard solvents on enantioselectivity and enantiomer elution order of ALA and DHALA on the Chiralpak IH-3 CSP. Chromatographic conditions as in Table 2.

**Figure 3:**
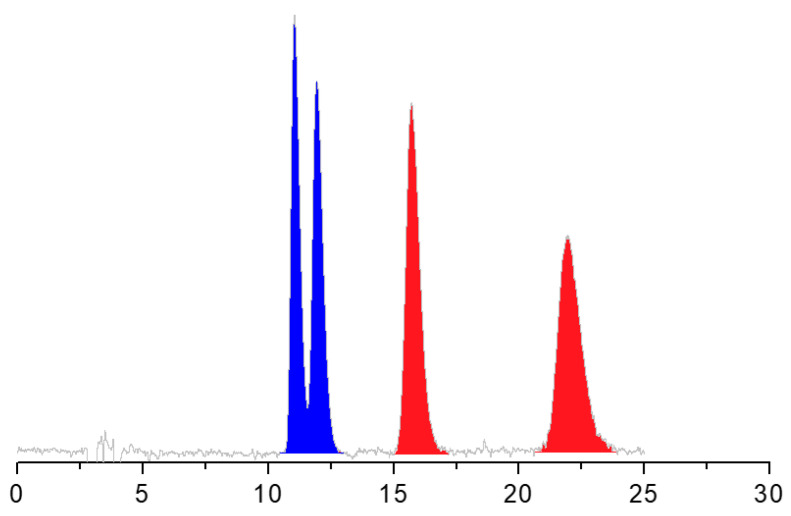
HPLC separation of enantiomers of ALA and DHALA on the Chiralpak AS-H CSP. Chromatographic conditions: column, Chiralpak AS-H (250 mm × 4.6 mm, 5 μm); mobile phase, n-hexane/IPA/TFA 80:20:0.1 (*v*/*v*/*v*); temperature, 5 °C; flow rate, 1 mL/min; detection, UV at 330 nm.

A close look on the chromatographic and thermodynamic data presented reveals that:

(i) All the analyzed enantioseparations were characterized by the terms ΔΔH° and ΔΔS° of same negative sign. According to these results, the enantiorecognition process was favored by the enthalpic contribution and disfavored by the entropic term.
(ii) The isoelution temperature values were, with a unique exception (entry 7, Table 2), higher than the explored range of temperature. Thus, when increasing the temperature within the enthalpy-controlled domain (|T_ISO_ΔΔS°| < |ΔΔH°|), no change in enantiomer elution order was observed. In the analysis of DHALA on the Chiralpak IH-3 with n-hexane/IPA/TFA 80:20:0.1 (*v*/*v*/*v*) (entry 7), a poor enantioseparation (α = 1.04) was achieved at 5 °C and above T_ISO_ (i.e., 25 °C); the two enantiomers coeluted and never split.
(iii) The enantioseparation factors of both acids progressively increased as the column temperature decreased and moved away from the T_ISO_ value. Examples of enantioseparations of ALA and DHALA on the Chiralpak IH-3 optimized at the temperature of 5 °C are shown in Figure 2.
(iv) The resolution factor values for DHALA obtained with non-standard mobile phases on the immobilized Chiralpak IH-3 (entries 8–10) were higher than those observed using the coated Chiralpak AS-H CSP in standard elution mode (entry 6).
(v) To conclude this section, it is also interesting to mention that the enantiomers of ALA were separated from those of DHALA only on the Chiralpak AS-H CSP. The simultaneous chemo- and enantioseparation is shown in Figure 3.

### 2.3. Absolute Configuration and Enantiomer Elution Order Determination

**Figure 4:**
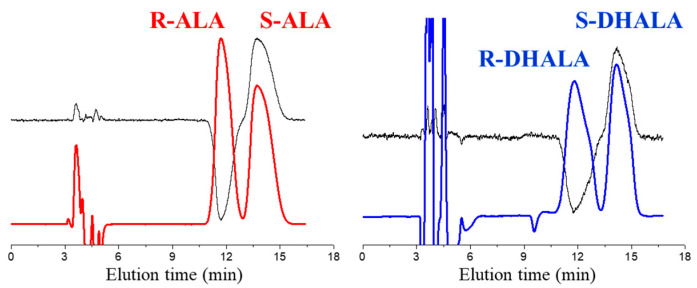
HPLC resolution of 0.25 mg of ALA and DHALA. Chromatographic conditions: column, Chiralpak IH-3 250 mm × 4.6 mm; mobile phase, n-hexane-2-propanol-TFA 80:20:0.1 (*v*/*v*/*v*) for ALA and n-hexane-2-propanol-DCM-TFA 80:5:15:0.1 (*v*/*v*/*v*/*v*) for DHALA; flow-rate, 1.0 mL/min; detection, CD (black) and UV at 330 nm.

In order to establish the enantiomeric elution order on the amylose-based CSPs used in this work, enantioenriched forms of ALA and DHALA (ee > 95%) were isolated by repetitive enantioseparations on the analytical 250 mm × 4.6 mm Chiralpak IH-3 column and submitted to chiroptical analysis. The mixtures selected as mobile phases for HPLC enantioseparations were: n-hexane-2-propanol-TFA 80:20:0.1 (*v*/*v*/*v*) for ALA (Entry 2, Table 2) and n-hexane-2-propanol-DCM-TFA 80:5:15:0.1 for DHALA (Entry 8, Table 2). In both cases, the column temperature was set at 5 °C (see Table 2 for other chromatographic conditions). Representative chromatograms for the resolution of 0.25 mg of racemic samples are shown in Figure 4.

**Figure 5:**
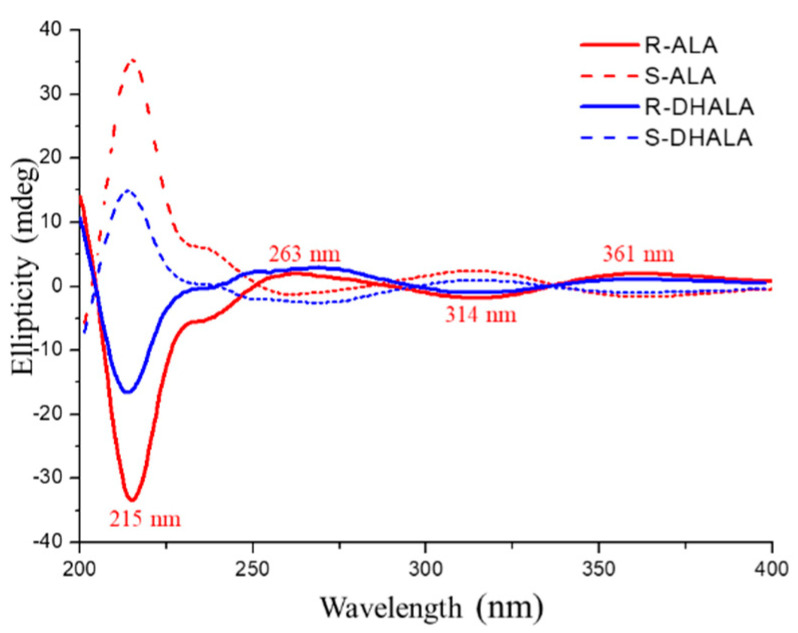
CD spectra of the enantiomers of ALA and DHALA in ethanol solution.

The evaluation of the sign of optical rotation of collected enantiomers was the empirical approach for the assignment of the absolute configuration of ALA. As previously reported [24], the (*R*)-ALA enantiomer, in ethanol solution, is dextrorotatory. The first eluted enantiomer of ALA on the Chiralpak IH-3 CSP had the same sign of optical rotation and, therefore, (*R*)-configuration. The CD spectra of enantiomers of the reference compound ALA were recorded and compared with those of enantiomers of DHALA. As seen in Figure 5, the CD spectrum of (*R*)-(+)-ALA exhibited two weak and broad positive bands located at 361 nm and 263 nm, alternating with a weak negative band at 314 nm and a more intense and sharper negative band centered at 215 nm. The same Figure 5 highlights how the less retained enantiomer of DHALA on the Chiralpak IH-3 CSP showed the same CD band sequence of R-ALA. Therefore, based on the close analogy in CD properties, it may be possible to empirically assign the (*R*)-configuration to the stereogenic C6 atom of the first eluted enantiomers of both chiral acids.

**Figure 6:**
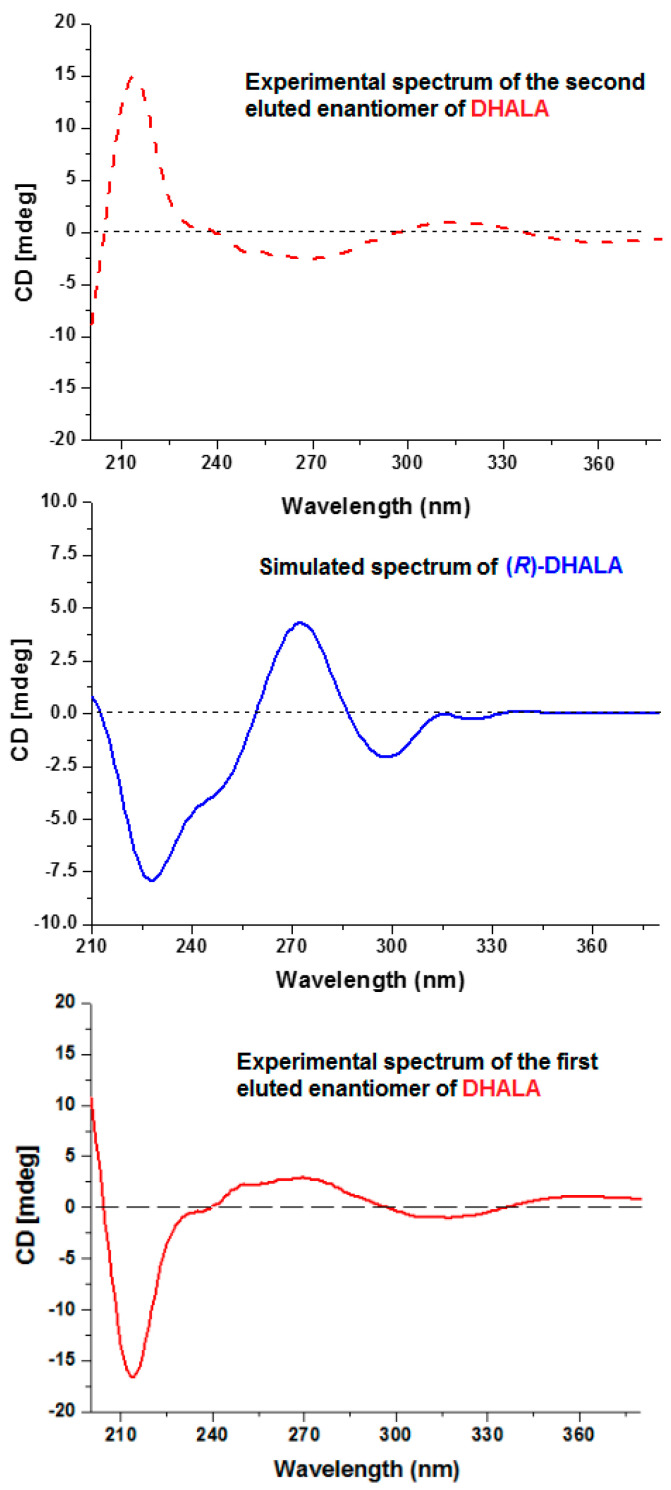
Experimental (full and dashed red lines) and calculated (blue line) CD spectra of R-DHALA.

In order to obtain a definitive confirmation about the absolute *R*-configuration above attributed by empirical considerations to the first chromatographically eluted enantiomer of both ALA and DHALA, we also performed DFT simulations of the CD spectrum [25,26,27] for (*R*)-DHALA (calculation details about such estimations are given within the Materials and Methods section). As visible in Figure 6, the comparison of such spectrum with the experimental ones affords further favorable support to the attribution of the *R*-configuration to the enantiomer of DHALA eluted as the first species from the columns Chiralpak AS-H and Chiralpak IH-3.

As can be seen from the data reported in Table 1, under each eluting condition explored in this study, the elution order of the enantiomers of ALA and DHALA was the same (i.e. the (*R*)-enantiomers were less retained than the (*S*)-enantiomers).

A further examination reveals that the CD sign exhibited by the (*R*)-enantiomer at the diagnostic wavelength of 330 nm was always negative. Therefore, the sign of CD peak is negative or positive as a function of the absolute configuration. Consequently, this univocal correlation can be used as a direct readout of the stereochemistry at C6 of ALA and DHALA.

## 3. Materials and Methods

### 3.1. Chemical and Reagents

ALA, DHALA, and the HPLC-grade solvents n-hexane, IPA, EtOH, DCM, EA, and THF were purchased from Sigma-Aldrich (Milan, Italy). MTHF was purchased from Carlo Erba (Milan, Italy). HPLC analyses were carried out on the Chiralpak IH-3 (250 mm × 4.6 mm, 3 µm) and Chiralpak AS-H (250 mm × 4.6 mm, 5 µm) columns (Chiral Technologies Europe, Illkirch-Graffenstaden, France). It is important to note that the impact of the particle size (3 µm and 5 µm) of two columns on the efficiency and resolution has not been evaluated.

### 3.2. Instruments and Chromatographic Conditions

The chromatographic system consisted of a UHPLC Jasco LC-4000 (Jasco, Tokyo, Japan) equipped with a 4095CD CD chiral detector. Data acquisition was performed using the Jasco ChomNAV software.

Fresh standard solutions of ALA and DHALA for HPLC analysis were prepared by dissolving the analytes in ethanol (conc. about 0.2 mg/mL). The injection volume was 50 μL.

The CD spectra were measured in a 0.1 cm path-length quartz cell at 25 °C using a Jasco Model J-700 spectropolarimeter. The spectra are average computed over four instrumental scans, and the intensities are presented in terms of ellipticity values (mdeg). Optical rotations were measured at 589 by a PerkinElmer polarimeter model 241 equipped with Na/Hg lamps. The volume of the cell was 1 mL, and the optical path was 10 cm. The system was set at a temperature of 20 °C.

### 3.3. Simulation of the Chiroptical Properties of (R)-DHALA Enantiomer Employed for the Assignment of the Absolute Configuration

Assignment of the absolute configuration to the first eluted peak of the species DHALA from the employed HPLC chiral columns can be summarized in the following steps:

(i) conduction of a conformational search based on molecular mechanic calculations (MM, force field: MMFF94) performed on a structure of DHALA characterized by (*R*)-configuration, followed by the selection of the more representative conformations inside an energetic window of 3.1 kcal mol^−1^ (10 conformations) corresponding to an overall Boltzmann population of 97%; (ii) further optimization at the AM1 semiempirical level of theory of the 10 geometries selected from the above MM conformational search, followed by the selection of the resulting 4 energetically more representative conformations (which correspond to an overall Boltzmann population of 77%); (iii) quantum-mechanical simulation of electronic CD assessed for the 4 conformations of (*R*)-configuration achieved in the previous second step (CD_1–4_). The final CD spectrum was then obtained by mixing of the 4 CD_1–4_ spectra, each of which weighted according to the respective Boltzmann population possessing the relevant conformation.

The quoted conformational search was carried out according to the systematic algorithm implemented in the computer program SPARTAN 10 v.1.1.0 (Wavefunction Inc., 18401 Von Karman Avenue, Suite 370, Irvine, CA 92612, USA). The conditions adopted in this analysis were: (a) all the rotatable bonds varied; (b) 40 kJ × mol^−1^ as maximum energy gap from the lowest energy geometry imposed for kept conformations; (c) *R*^2^ ≥ 0.9, the criterion adopted to define conformers as duplicates in the analysis of similarity between conformations. Similarly, the above mentioned energy optimization steps concerning the sets of selected structures were also carried out by means of the program SPARTAN 10 v.1.1.0.

The simulations of CD spectra were performed through the algorithms implemented in the Amsterdam Density Functional (ADF) package v. 2007.01. The options set for such calculations were:

- single point at the BLYP level of theory, employing the TZ2P Large Core basis set;
- ethanol as the solvent;
- 50 singlet excitations; diagonalization method: Davidson; velocity representation; scaling factor 1.3; peak width 20.0.

## 4. Conclusions

In this work it has been demonstrated that the amylose-based Chiralpak AS-H and Chiralpak IH-3 CSPs provide direct and complete resolution of ALA-enantiomers under normal-phase conditions. Optimization of chiral discrimination was achieved at 5 °C. The solvent versatility of the Chiralpak IH-3 CSP was exploited to set up baseline enantioseparation of DHALA using non-standard mobile phases containing DCM, EA, and the eco-compatible solvent MTHF as an organic modifier. The proposed HPLC methods can be regarded as useful tools to assess the enantiomeric composition of both chiral acids in natural and synthetic samples and to investigate the pharmacodynamic/pharmacokinetic/toxicity properties of single enantiomers.

## Figures and Tables

**Table 1:** Chromatographic results in normal-phase conditions.

Compound	CSP	Mobile Phase	*k*_1_ (AC)-(CD) ^1^	α	*Rs*
ALA	AS-H	n-Hexane-IPA-TFA 80:20:0.1	2.06 (*R*)-(-)	1.29	3.14
	IH-3	n-Hexane-IPA-TFA 80:20:0.1	1.52 (*R*)-(-)	1.16	2.77
	AS-H	n-Hexane-EtOH-TFA 85:15:0.1	1.18 (*R*)-(-)	1.11	1.23
	IH-3	n-Hexane-EtOH-TFA 85:15:0.1	1.19 (*R*)-(-)	1.08	1.19
	IH-3	n-Hexane-IPA-DCM-TFA 80:5:15:0.1	2.23 (*R*)-(-)	1.13	1.89
	IH-3	n-Hexane-IPA-EA-TFA 90:5:5:0.1	2.69 (*R*)-(-)	1.09	1.75
	IH-3	n-Hexane-IPA-THF-TFA 80:5:15:0.1	1.09 (*R*)-(-)	1.07	<1
	IH-3	n-Hexane-IPA-MTHF-TFA 80:5:15:0.1	1.54 (*R*)-(-)	1.10	1.12
DHALA	AS-H	n-Hexane-IPA-TFA 85:15:0.1	1.43 (*R*)-(-)	1.06	<1
	IH-3	n-Hexane-IPA-TFA 85:15:0.1	1.18 (*R*)-(-)	1.00	-
	AS-H	n-Hexane-EtOH-TFA 85:15:0.1	0.89 (*R*)-(-)	1.00	-
	IH-3	n-Hexane-EtOH-TFA 85:15:0.1	1.22 (*R*)-(-)	1.00	-
	IH-3	n-Hexane-IPA-DCM-TFA 80:5:15:0.1	2.32 (*R*)-(-)	1.14	2.88
	IH-3	n-Hexane-IPA-EA-TFA 90:5:5:0.1	2.79 (*R*)-(-)	1.11	2.85
	IH-3	n-Hexane-IPA-THF-TFA 80:5:15:0.1	1.11 (*R*)-(-)	1.07	<1
	IH-3	n-Hexane-IPA-MTHF-TFA 80:5:15:0.1	1.67 (*R*)-(-)	1.10	1.89

^1^ Absolute configuration (AC) and sign of the dichroism circular (CD) at 330 nm referred to the first eluted enantiomer. Chromatographic conditions: column, Chiralpak AS-H (250 mm × 4.6 mm, 5 μm) and Chiralpak IH-3 (250 mm × 4.6 mm, 3 μm); flow rate, 1 mL/min; temperature, 25 °C; detection, UV and CD at 330 nm. ALA: α-lipoic acid; DHALA: α-dihydrolipoic acid; IPA: 2-propanol; EtOH: ethanol; DCM: dichloromethane; EA: ethyl acetate; THF: tetrahydrofuran; MTHF: 2-methyltetrahydrofuran.

**Table 2:** Retention and enantioseparation factors at 5 °C and thermodynamic data of ALA and DHALA.

Entry	Compound	CSP	Mobile Phase	α (5°C)	Rs (5°C)	ΔΔH*°* (kcal/mol)	ΔΔS*°* (e.u.)	T_ISO_ (°C)
1	ALA	AS-H	n-Hexane-IPA-TFA 80:20:0.1	1.49	4.62	−1.23	−3.61	68
2		IH-3	n-Hexane-IPA-TFA 80:20:0.1	1.22	3.16	−0.36	−0.95	108
3		IH-3	n-Hexane-IPA-DCM-TFA 80:5:15:0.1	1.16	2.80	−0.31	−0.78	116
4		IH-3	n-Hexane-IPA-EA-TFA 90:5:5:0.1	1.17	2.80	−0.13	−0.26	230
5		IH-3	n-Hexane-IPA-MTHF-TFA 80:5:15:0.1	1.12	1.67	−0.21	−0.53	127
6	DHALA	AS-H	n-Hexane-IPA-TFA 80:20:0.1	1.11	1.24	−0.40	−1.24	52
7		IH−3	n-Hexane-IPA-TFA 80:20:0.1	1.04	<1	−0.30	−1.00	25
8		IH-3	n-Hexane-IPA-DCM-TFA 80:5:15:0.1	1.19	3.95	−0.38	−1.03	97
9		IH-3	n-Hexane-IPA-EA-TFA 90:5:5:0.1	1.17	3.38	−0.32	−0.86	100
10		IH-3	n-Hexane-IPA-MTHF-TFA 80:5:15:0.1	1.13	2.56	−0.27	−0.72	102

Chromatographic conditions: column, Chiralpak AS-H (250 mm × 4.6 mm, 5 μm) and Chiralpak IH-3 (250 mm × 4.6 mm, 3 μm); flow rate, 1 mL/min; detection, UV and CD at 330 nm. IPA: 2-propanol; EtOH: ethanol; DCM: dichloromethane; EA: ethyl acetate; THF: tetrahydrofuran; MTHF: 2-methyltetrahydrofuran.

## Data Availability

Data sharing not applicable.

## Sample Availability

Samples of the compounds ALA and DHALA are available from the authors.
